# POCD in patients receiving total knee replacement under deep vs light anesthesia: A randomized controlled trial

**DOI:** 10.1002/brb3.910

**Published:** 2018-01-30

**Authors:** Ruixue Hou, Hong Wang, Lianhua Chen, Yimin Qiu, Shitong Li

**Affiliations:** ^1^ Department of Anesthesiology Shanghai General Hospital Affiliated to Shanghai Jiaotong University Shanghai China

**Keywords:** general anesthesia, nerve block, postoperative cognitive dysfunction

## Abstract

**Objectives:**

Clinical observation, as well as randomized controlled trials, indicated an increasing rate of postoperative cognitive dysfunction (POCD) with increasing depth of general anesthesia. However, the findings are subject to bias due to varying degree of analgesia. In this trial, we compared the rate of POCD between patients receiving light versus high anesthesia while holding analgesia comparable using nerve block.

**Methods:**

Elderly patients (≧60 years) receiving elective total knee replacement were randomized to receive the surgery under general anesthesia at BIS 40–50 (LOBIS group) or BIS 55–65 (HIBIS group). The femoral nerve and the sciatic nerve were blocked under ultrasonic guidance in all patients before induction. Cognitive performance was assessed with Montreal cognitive assessment (MoCA) at the baseline and 1d, 3d, and 7d after the surgery. POCD was defined by Z score of >1.96 using cross‐reference. The extubation time and recovery time were also compared.

**Results:**

A total of 66 patients were randomized; 60 (*n* = 30 per group) completed trial as the protocol specified. POCD occurred in six patients (20%) in the LOBIS group vs. in one patient (3.3%) in the HIBIS group (Figure 3, *p* = .04). In all seven cases, the diagnosis of POCD was based on MoCA assessment on 1d after the surgery. Assessment in 3d and 7d after surgery did not reveal POCD in any case. Extubation time was longer in the LOBIS group (12.16 ± 2.58 vs. 5.77 ± 3.01 min in the HIBIS group (*p < *.001)). The time of comeback of directional ability was 13.47 ± 3.14 and 6.17 ± 3.23 min in the LOBIS and HIBIS groups, respectively (*p < *.001).

**Conclusions:**

In elderly patients receiving a total knee replacement, lighter anesthesia could reduce the rate of POCD with complete analgesia during surgery.

## 1 INTRODUCTION

Postoperative cognitive dysfunction (POCD) is typically transient, but could be associated with increased incidence of permanent dementia and even mortality (Newman et al., 2001; Steinmetz, Christensen, Lund, Lohse, & Rasmussen, 2009). A number of factors, including the type and depth of anesthesia, contribute to the development of POCD (Chan, Cheng, Lee, & Gin, 2013; Farag, Chelune, Schubert, & Mascha, 2006; Steinmetz, Funder, Dahl, & Rasmussen, 2010). Farag et al. (2006) believed that deeper anesthesia could reduce the rate of POCD by decreasing cerebral metabolism and preventing overt responses to noxious stimuli. In contrast, Chan et al. (2013) showed that BIS‐guided lighter anesthesia improves early postoperative recovery. Regardless of the nature of the association with anesthesia depth, perioperative pain plays a pivotal role in the development of POCD (Chi et al., 2013; Degos et al., 2013). It would be inappropriate to discuss the relationship between the depth of anesthesia and POCD ignoring the interference of analgesic on the result. In this study, we examined the rate of POCD in elderly patients receiving total knee replacement under deep vs. light anesthesia while holding analgesia comparable using nerve block.

## 2 MATERIAL AND METHODS

This study was approved by the Ethics Committee of Shanghai General Hospital Affiliated to Shanghai Jiaotong University and registered with Chinese Clinical Trials.gov (ChiCTR‐INR‐17010713). Written informed consent was obtained from all participants. Subjects receiving elective total knee arthroplasty were randomized as per random number to receive anesthesia at either a deep level (BIS at 40–50; the LOBIS group) or a light level (BIS at 55–65; the HIBIS group). Inclusion criteria included: (i) ASA I or II; (ii) age at ≧60 years. Exclusion criteria included: (i) severe cardiovascular diseases, diabetes mellitus, neurological or psychiatric illnesses, hepatic and/or kidney dysfunction; (ii) regular use of analgesics or antidepressants prior to the surgery; (iii) cognitive dysfunction, as defined as Montreal cognitive assessment (MoCA) at <23, prior to the surgery; and (iv) inability to comply with the study protocol or procedures.

Prior to anesthesia induction, nerve block was carried out under ultrasonic guidance. The femoral and the sciatic nerve were blocked with 10‐ml 0.375% ropivacaine in solution with 10‐ml 1% lidocaine. The sciatic nerve was blocked using a modified lateral approach (from the upper edge of the patella and the tendon of the biceps femoris). After 20 min, the loss of pinprick sensation (VAS<3) was verified using a short‐beveled 25‐gauge needle. Anesthesia was induced with 0.02 mg/kg midazolam, 1.5 μg/kg fentanyl, 1.5 mg/kg propofol, and 0.6 mg/kg rocuronium. Mechanical ventilation was carried out by volume‐controlled mode (tidal volume 8 ml/kg, I:E = 1:1.5, PetCO_2_ 30–40 mmgH) using a laryngeal mask. Anesthesia was maintained with sevoflurane (MAC 0.3) in combination with propofol at varying infusion rate to either BIS 55–65 (HIBIS group) or BIS 40–50 (LOBIS group). Upon completion of the surgery, neostigmine was used to reverse neuromuscular blockade. Subjects were transferred to the postoperative care unit after extubation.

Blood pressure was managed using a routine protocol. Ephedrine was given at a bolus of 6 mg if mean blood pressure (MBP) decreases to <80% of the preoperative baseline or systolic blood pressure decreases to <90 mmHg; atropine (0.3 mg) was given if HR decreases to <50 beats/min. If MBP or HR increases by >20% of the preoperative baseline, patients received fentanyl (0.05 mg) followed by perdipine (0.5 mg) or esmolol (1 mg).

**Figure 1:**
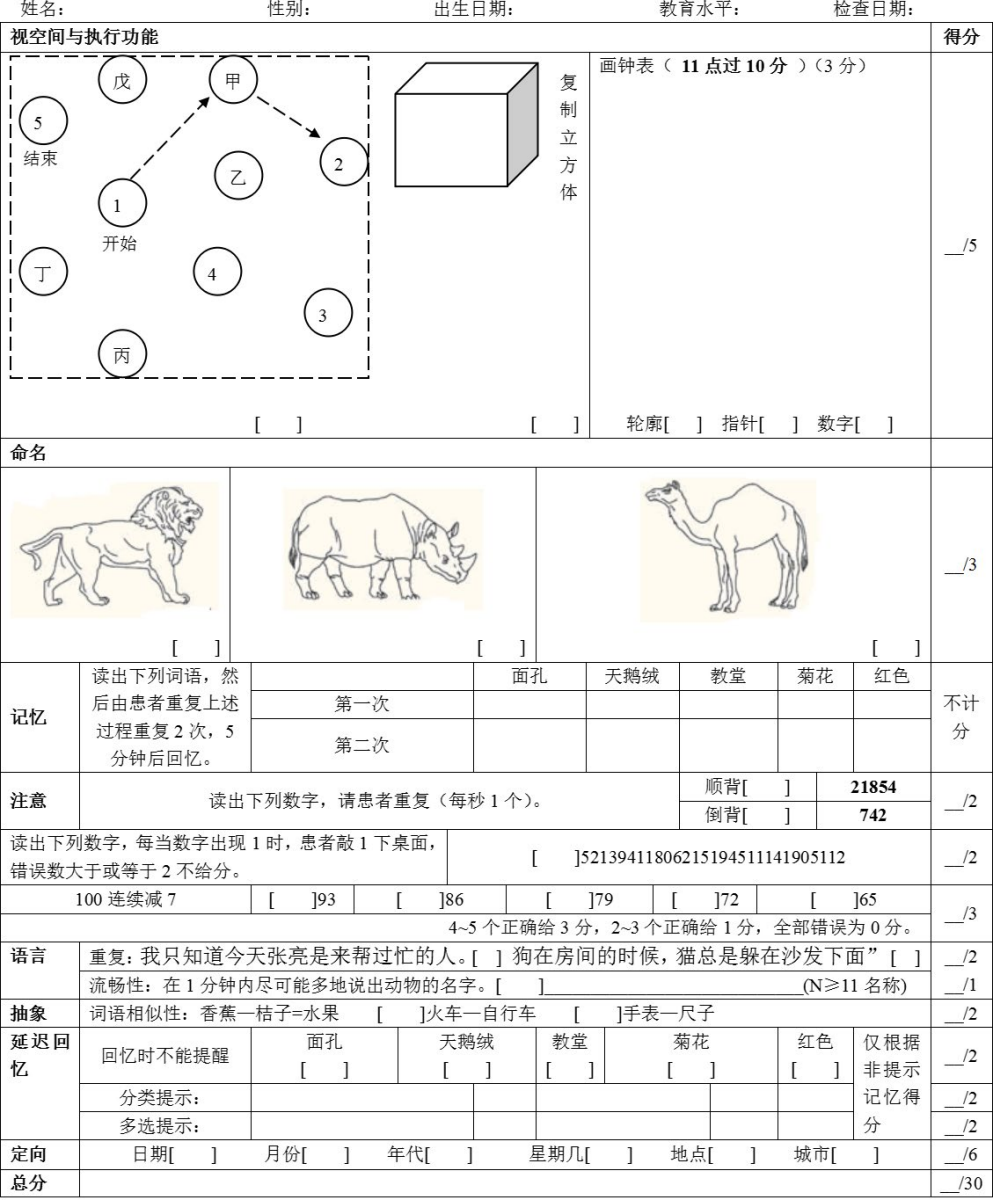
Montreal cognitive assessment (MoCA) Beijing version

In addition to the baseline, the neuropsychological assessment was conducted at 1d, 3d, and 7d after the surgery using Montreal cognitive assessment (MoCA) by an experienced psychiatrist. The MoCA assessment included 16 items and 11 categories, and examines visuospatial and executive functions, naming, memory, attention, language, abstraction, and orientation (Figure 1). POCD was defined as Z score at >1.96, as previously described (Buvanendran et al., 2006; Chi et al., 2013). The study is based on cross‐reference: Z score in the HIBIS group was calculated using the LOBIS group as reference, and vice versa.

The pain was assessed using a 0–10 visual analogue scale (VAS; 0 =  no pain and 10 =  worst pain imaginable). Postoperative analgesia was regularly conducted using patient‐controlled analgesia (PCA): Sufentanil was delivered at a rate of 0.15 μg/hrs, with 1.5‐μg bolus and lockout interval of 15 min for breakthrough pain. Nonopioid pain medication was added by nursing staff if visual analogue scale (VAS) >4. Moreover, other parameters were also recorded including hemodynamic variables, extubation time, recovery time, the duration of surgery, and others.

### 2.1 Statistical analysis

The sample size requirement was estimated based on the following assumptions: (i) ɑ at 0.05; (ii) 1 – β at 0.80; (iii) POCD rate at 41% in the LOBIS group, based a previous report in elderly patients receiving total knee replacement by Rodriguez et al. (2005), and a clinically meaningful reduction in POCD rate at 15%. The calculation yielded 23 participants per group. Considering the possibility of attrition, we planned to enroll a total of 66 subjects.

All statistical analyses were conducted using the SPSS 19.0 software. All continuous variables were normally distributed (as verified by the Shapiro–Wilk method), analyzed with Student's *t* test, and presented as mean ± standard deviation. Categorical data were analyzed using chi‐squared test or Fisher's exact test. Statistical significance was set at *p *<* *.05.

## 3 RESULTS

**Figure 2:**
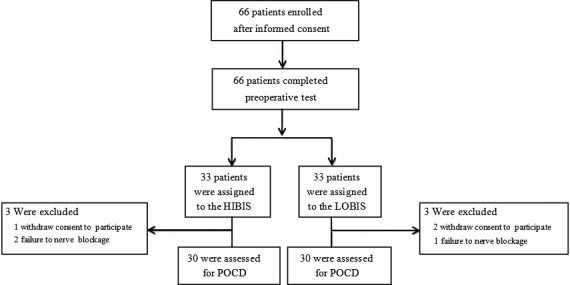
Flow diagram of participants

A total of 66 subjects were randomized (Figure 2). Sixty subjects completed the trial as planned (*n* = 60 in each group); the remaining six did not complete the trial due to either unsuccessful nerve block (*n* = 3) or unavailable MoCA data (*n* = 3). The difference in dropout rate between the two groups was not statistically significant (*p *>* *.05). BIS score was 62.63 ± 1.49 and 41.80 ± 2.01 in the HIBIS and LOBIS groups, respectively (Table 1). No subject reported intra‐operative awareness. Baseline characteristics, including demographic data and length of the surgical operation, were generally comparable between the two groups (Table 1).

**Table 1:** Participant characteristics and anesthetic variables

	HIBIS (*n* = 30)	LOBIS (*n* = 30)
Age (year)	68.5 ± 7.9	67.9 ± 6.5
Height (cm)	159.4 ± 6.7	161.4 + 6.5
Weight (kg)	65.3 ± 6.1	67.9 ± 6.9
Sex (male/female)	12/18	14/16
Education (year)	7.8 ± 2.7	7.1 ± 2.4
Length of surgery (min)	74.7 ± 15.7	69.9 ± 12.7
Propofol dose (mg/kg/hrs)	2.88 ± 0.60	4.56 ± 1.20^a^
Fentanyl dose (mg)	5.79 ± 1.20	4.74 ± 3.00
Time of extubation (min)	5.77 ± 3.01	12.16 ± 2.58^a^
Time of recovery (time)	6.17 ± 3.23	13.47 ± 3.14^a^
Average BIS	62.63 ± 1.49	41.80 ± 2.01^a^

a *p *<* *.05 versus Group HIBIS.

**Figure 3:**
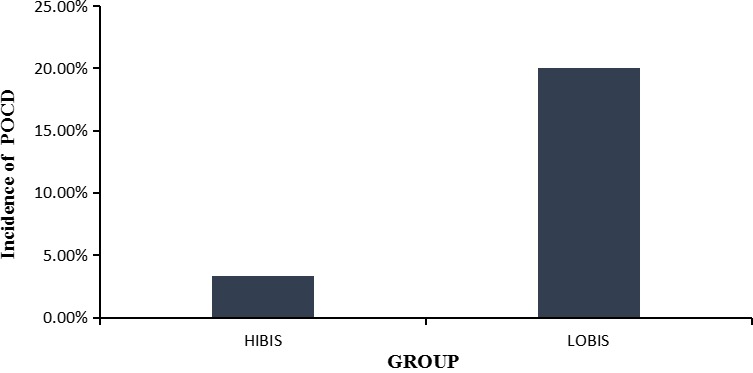
The incidence of POCD between two groups

POCD occurred in six patients (20%) in the LOBIS group vs. in one patient (3.3%) in the HIBIS group (Figure 3, *p* = .04). In all seven cases, POCD was based on MoCA assessment on 1d after the surgery. Assessment in 3d and 7d after surgery did not meet POCD definition in any case. The VAS pain score did not differ significantly between the two groups at any time point (Table 2). No subject required additional analgesic.

**Table 2:** Postoperative pain scores using the visual analogue scale (VAS)

Group	POD‐1	POD‐3	POD‐7
HIBIS	3.70 ± 1.18	0.93 ± 0.64	0.13 ± 0.35
LOBIS	3.56 ± 0.86	1.10 ± 0.99	0.20 ± 0.41

POD, postoperative day.

Propofol infusion rate was 2.88 ± 0.6 and 4.56 ± 1.2 mg/kg/hrs in the HIBIS and LOBIS groups, respectively (*p *<* *.001). Total dosage of fentanyl during the surgery was 5.79 ± 1.2 and 4.74 ± 3.0 mg in the HIBIS and LOBIS groups, respectively (*p = *.09). Extubation time was longer in the LOBIS group (12.16 ± 2.58 vs. 5.77 ± 3.01 min in the HIBIS group, *p *<* *.001). The time of comeback of directional ability was 13.47 ± 3.14 min in the LOBIS group and 6.17 ± 3.23 min in the HIBIS group (Table 1, *p* < .001). Hemodynamic profiles were comparable between the two groups (Table 3).

**Table 3:** Changes of hemodynamics between two groups

		Before induction	After induction	Before intubation	After intubation	Before extubation	After extubation
MAP (mmgH)	I	86.27 ± 2.92	76.27 ± 4.24	80.00 ± 3.64	83.50 ± 3.35	86.77 ± 2.66	87.17 ± 3.23
	II	85.00 ± 2.52	77.63 ± 4.92	83.03 ± 3.88	83.13 ± 3.44	86.48 ± 2.33	86.47 ± 3.39
HR (bpm)	I	71.97 ± 4.69	71.47 ± 3.46	70.50 ± 4.06	73.13 ± 4.64	72.27 ± 4.71	71.73 ± 4.59
	II	73.47 ± 3.46	70.3 ± 4.16	70.3 ± 3.64	72.67 ± 4.33	70.03 ± 3.83	69.77 ± 3.54

## 4 DISCUSSION

The current study indicated that lighter anesthesia with complete analgesia through nerve block results in lower rate of early POCD. Extubation time could be decreased. Patient recovery could be expedited. The rate of POCD in the HIBIS group of the current study (3.3%) is much lower than the reported rate in patients receiving noncardiac surgical procedures (25%–44%) (Li, Wen, Zhao, Hang, & Mandell, 2013; Moller et al., 1998; Monk & Price, 2011; Price et al., 2014). Such a difference could be attributed to differences of a variety of factors, including the definition of POCD, surgery type, and patient demographic features. Regardless, our results contradicted with the notion by Farag et al. (2006) and supported a higher rate of POCD in patients receiving deeper anesthesia.

Perioperative pain could lead to cognitive dysfunction after anesthesia and surgery (Lin et al., 2012; Zhang et al., 2013). In the current study, we excluded the potential bias caused by inadequate analgesia by nerve blockage in all patients prior to the surgery. Postoperative pain was also controlled by PCA and additional nonopioid agents at VAS at <3 after surgery.

The computer‐generated BIS is a noninvasive and objective indicator of anesthesia depth (Iselinchaves, Moalem, Gan, Ginsberg, & Glass, 2000; Sprung et al., 2000). In most type of surgeries, the BIS target range is set at 40–60 (Johansen & Sebel, 2000; Johansen, Sebel, & Sigl, 2000). BIS values at <40 are linearly correlated with burst suppression ratio and increase mortality (Monk, Saini, Weldon, & Sigl, 2005). Therefore, we set the minimum limit of BIS value to 40 in the LOBIS group. The US FDA recommends BIS 40–60 to prevent intra‐operative awareness. In the current study, we set the maximum BIS at 65 in the HIBIS group. Such a design was based on the use of nerve block that decreases the transmission of peripheral stimuli to the central nervous system, and the higher threshold for arousal with the use of a laryngeal mask. No subject in the current study reported intra‐operative awareness, suggesting the BIS 65 in patients with nerve block is practical.

Propofol infusion rate in the current study (2.88 ± 0.60 and 4.56 ± 1.20 mg/kg/hrs in the HIBIS and LOBIS groups, respectively) was much lower than reported when general anesthesia is used alone (6–10 mg/kg/hrs). It is consistent with previous studies showing that a combination of a regional nerve block with general anesthesia could decrease the dosage of anesthetic agents (Junger et al., 2001; Wennervirta et al., 2008). In our study, anesthesia was maintained with sevoflurane (MAC 0.3) in combination with a syringe‐pump infusion of propofol. Such a design was based on the fact that propofol can be readily adjusted to achieve and maintain BIS at the target range (Wysowski & Pollock, 2006). Also, previous studies reported a higher incidence of intra‐operative awareness with total intravenous anesthesia (Ghoneim, Block, Haffarnan, & Mathews, 2009; Pollard, Coyle, Gilbert, & Beck, 2007).

Possible contribution by fentanyl to POCD is unknown in the current study. In a previous study of 326 elderly patients undergoing cardiac surgery by Silbert et al. (2006), high‐dose fentanyl (50 μg/kg) is not associated with a difference of the rate of POCD compared with low‐dose fentanyl (10 μg/kg) at 3–12 months after surgery. However, the low‐dose fentanyl group had higher short‐time (1‐week) rate of POCD (OR, 2.15; *p *=* *.025). They speculated that such a result reflects the effect of high‐dose fentanyl in inhibiting stress response. Collectively, these results encourage lighter anesthesia with complete analgesia in elderly patients.

The sample size of the current study is relatively small. Also, MoCA testing is only one tool to assess POCD. Accordingly, the results obtained with the current study cannot be directly compared with other studies. Also, we did not examine pro‐inflammatory cytokines levels in blood samples between the two groups. We are currently planning studies to address these issues.

## 5 CONCLUSIONS

In elderly patients undergoing total knee replacement, lighter anesthesia (BIS 55–65) with complete analgesia through nerve block could decrease the development of POCD.

## CONFLICT OF INTEREST

There is no conflict of interest related to this work.
